# Understanding Hesitation to Use Nicotine Replacement Therapy: A Content Analysis of Posts in Online Tobacco-Cessation Support Groups

**DOI:** 10.1177/08901171221113835

**Published:** 2022-07-11

**Authors:** Connor Phillips, Cornelia Pechmann, Douglas Calder, Judith J Prochaska

**Affiliations:** 1 Paul Merage School of Business, 8788 University of California Irvine, Irvine, CA, USA; 2 Stanford Prevention Research Center, Department of Medicine, 6429 Stanford University, Stanford, CA, USA

**Keywords:** smoking cessation, tobacco use, nicotine replacement therapy, adherence, compliance, tobacco control

## Abstract

**Purpose:**

We aimed to better understand hesitancy to use nicotine replacement therapy (NRT) to quit smoking.

**Design:**

We content coded and analyzed NRT-related posts in online quit smoking support groups to understand NRT-use hesitancy and to examine associations with health outcomes.

**Setting:**

NRT posts were analyzed in unmoderated social-media support groups with free NRT.

**Sample:**

Adults who smoked daily (n = 438) and posted about NRT were studied, 339 of whom reported on NRT usage and 403 reported on smoking abstinence.

**Measures:**

Surveys at 1-month post-quit date assessed NRT usage and smoking abstinence.

**Analysis:**

Relationships among NRT posts, NRT usage and smoking abstinence were analyzed using GEE models accounting for support group and covariates.

**Results:**

Nearly all (96.17%) participants reported using the study-provided NRT once, most (70.21%) used NRT during the past week, but less than half (45.72%) used NRT daily for the full month as recommended. Nearly two-thirds (65.34%) of NRT posts were negative. Posts reflecting dislike or no longer needing NRT were associated with a lower likelihood of using NRT in the past week at least once (B = −.66, *P* = .005 and B = −.37, *P* = .045), use occasions (B = −1.86, *P* = .018 and B = −1.10, *P* = .016) and used daily for full month (B = −.56, *P* = .044 and B = −.53, *P* = .009). Posts related to the effectiveness of NRT related to past-week NRT used at least once (B = .15, *P* = .023), used daily for full month (B = .25, *P* = .001), and smoking abstinence (B = .27, *P* = .002).

**Conclusion:**

Strategies are needed to address dislike of NRT and strengthen perceptions of NRT efficacy, especially on social media where posts may be amplified.

## Purpose

Nicotine replacement therapy (NRT), available in the form of transdermal patch, gum, lozenge, inhaler, nasal spray, or mouth spray (available outside of the US), is an effective method for promoting tobacco cessation.^1^ Use of NRT increases the likelihood of quitting smoking by 50%-70%.^1,2^ Combining NRT use with counseling or a support group can also double the chances of quitting smoking.^3^ Providing NRT free of charge to tobacco-cessation program participants increases utilization of NRT.^4^ Hence, tobacco-cessation studies often provide free NRT to improve outcomes and as an incentive for enrollment, because NRT is costly, averaging $185 per quit attempt.^5^ Nicotine replacement therapy is more effective in helping people quit smoking when used for at least 4 weeks,^6^ and NRT manufacturers recommend 8-12 weeks.^7^ Nicotine replacement therapy adherence, however, may vary among participants, especially because many tobacco-cessation programs are now administered remotely, eg, via quitlines, text messages or web programs.^8–10^ Studies providing free NRT as the main quit strategy are dependent on the frequency and duration of NRT usage by participants.

Eight tobacco-cessation studies were identified that provided free NRT and reported on participants’ NRT usage (see Online Appendix A).^11-18^ In each study, the treatment protocol called for daily NRT use; treatment length varied and averaged 4.5 weeks (SD 2.8). Assessments varied and ranged from 1 to 7 months. Participants’ NRT usage was reported as any product use or the number of days or times the product was used in different time periods.^11-18^ The most comprehensive measures assessed both the frequency and duration of usage across different time periods.

In the 8 studies, most participants reported using the free NRT at least once, but their adherence with the recommended medical regimen of daily NRT use for the entire treatment period was much lower, especially if the treatment lasted over 2 weeks. For instance, Cummings et al^6^ found that 78% - 89% of smokers used the free NRT at least once and, at 1 week, their average usage was 5 days out of 7; but, at 6 weeks, their average usage was only 21 days out of 42. Hajek et al^9^ found that smokers’ average usage of free NRT at 1 month was 24 days out of 30. Kerr et al^7^ reported that at one month, just 44% of smokers had used all their free NRT as directed.^7^ At 2 months or later, just 11%-20% of smokers had done so, according to both Kushnir et al^19^ and Voci at al.^12^

A study by Pearson et al^20^ sought to understand NRT non-adherence by examining smokers’ general perceptions of NRT and their NRT usage. Social media posts about NRT in an online tobacco-cessation community were examined and coded as extremely negative to extremely positive. Positive NRT posts correlated with NRT usage, but only among participants who procured the NRT at their own cost. Among participants who were given free NRT, there was no correlation between NRT posts and NRT usage.

In the current study, we aimed to better understand hesitancy to use NRT when provided freely. We conducted a content analysis of support-group participants’ posts about NRT received for free in a clinical trial. We coded for different types of positive and negative content, and then we related NRT post content to NRT usage and smoking abstinence. We studied any NRT usage through to daily NRT use for the entire month (ie, full adherence). The current analysis extends our prior work reporting on social media posts during quit efforts, but with a focus here on participants’ NRT-related posts.^19^

## Methods

### Sample

Our initial sample consisted of 720 treatment participants assigned to unmoderated, peer-to-peer online support groups for tobacco cessation in a randomized controlled trial.^21^ All participants gave online, written informed consent. These support groups communicated on social media, in private Twitter groups. Screening criteria for trial participation included being ages 21-59, currently smoking at least 5 cigarettes per day and hence eligible for NRT, intention to quit smoking in the next 30 days, English speaking, owning a mobile phone with unlimited texting and internet, and being active on social media.^21^ Participants were recruited primarily through a Facebook campaign that ran in all continental U.S. zip codes.^22^ In addition, separate Facebook campaigns ran in zip codes with high smoking expenditures and high prevalence of Black/African American or Hispanic/Latino residents to enhance participant diversity. We analyzed treatment participants’ social media posts about the free NRT they were given.

Of the 720 participants assigned to tobacco-cessation support groups, we identified an analysis sample of 438 participants (60.8%) who had posted to their group at least once about NRT. We focused our analyses on these 438 participants because their NRT posts served as our primary independent variable. Analysis sample participants did not significantly differ from non-analysis sample participants on any measured demographics. Participants randomized to the control condition (n = 240), who had no support groups and no observable social media posts, could not be included in our analysis of NRT-related posts.

### Intervention

The online support groups for tobacco cessation in the randomized controlled trial^21^ consisted of 36 groups, each with 20 adults who smoked daily and wanted to quit. The support groups were either co-ed or women-only. Each group had a 3-month duration and participants were instructed to set a quit date and quit by day 11. Twitter was used as the social media platform to utilize its application programming interface but posts were not public; instead, each group was set up to be private, meaning only group members could view the posts.^21^ Participants were assigned a tobacco-cessation buddy within the group and were encouraged to post to their buddy and others in their group at least daily. Tobacco-cessation discussion topics were posted to the group daily and National Cancer Institute’s smokefree.gov Website links were emailed weekly. Nicotine replacement therapy-related discussion topics were posted to the groups on days 16, 18, 24, and 37 and emails with an NRT usage guide and links to smokefree.gov NRT-related information were sent on days 4, 5, 9, and 19 (detailed in Appendix B). Virtually all of this information stressed NRT efficacy except our day 4 usage guide described below.

Prior to study start, participants were mailed free NRT that consisted of an 8-week supply of nicotine patches and either nicotine gum or lozenges. Participants’ NRT dosage was based on their pre-study tobacco use. If a participant smoked 10 or more cigarettes daily pre-study, the initial (4 week) patch dosage was 21 mg tapering to 14 mg and then 7 mg (2 weeks each); while for those smoking fewer than 10 cigarettes the initial (6 week) patch dosage was 14 mg tapering to 7 mg (2 weeks). Participants were also sent 420 pieces of nicotine gum, in 4 mg or 2 mg strength, stronger if they smoked their first cigarette sooner after waking.^23^ Participants could alternatively opt to receive 432 nicotine lozenges at 1 mg strength. The recommended NRT dosages were specified in the package inserts from the manufacturers (Habitrol or Nicorette) which were in the participants’ mailers and online on our website; and our website had a separate section explaining both dosage and usage; however, participants could modify their NRT dosage if they so desired.

As participants were encouraged to meet one other and prepare to quit on days 1-3, and then set a quit date and start using NRT between days 4-11, we emailed them a detailed NRT usage guide on day 4. This email is included in full in Online Appendix C.

In addition to this information, if a participant reached out to us with any NRT concerns, we emailed them suitable pre-written responses, which we needed to address concerns about NRT product quality, patches not sticking, dosage questions, skin or other reactions, and concerns about nicotine addiction (Appendix C). Furthermore, the manufacturers’ package inserts were included in the NRT products mailed to participants, and digital versions of the package inserts were displayed on our website. Additionally on our website, we included a video explaining how to chew and park nicotine gum,^24^ and information borrowed from the Habitrol website on how to apply nicotine patches and use nicotine gum or lozenges.

### Measures

We assessed whether NRT was used at least once in the past week, past-week NRT use occasions, daily use for a full month, and smoking abstinence. We used web-based surveys administered one month after each participant’s stated quit date. Survey non-respondents were contacted daily for several weeks via multiple channels to try to obtain their responses. To measure any use of NRT and use occasions in the past week, we asked: “Over the past 7 days, how many times did you use FDA approved NRT (eg, patches, gum, spray, lozenges, etc.)?” To measure daily use for the full month we asked: “How many days did you use FDA approved NRT (eg, patches, gum, spray, lozenges, etc.) since your quit date?” and determined whether NRT was used 28+ days. A participant was recorded as missing data on NRT usage if they failed to respond to these usage questions.

To measure smoking abstinence, participants were asked: “Over the past 7 days, how many cigarettes have you consumed?” “Over the past 7 days, how many times did you use tobacco products other than cigarettes, eg, cigars, pipe, snuff, chew, snus, or hookah?” “Over the past 7 days, how many times did you use an Electronic Nicotine Delivery System (ENDS) (eg, e-cigarettes, vape)?” A participant was recorded as abstinent if they reported no use of cigarettes, other tobacco products or ENDS; if any use, they were reported as non-abstinent. A participant was recorded as missing data on abstinence if they failed to respond to these questions.

### Coding of NRT Posts

We analyzed the first 45 days of posts to the online social media-based support groups for tobacco cessation. There were no NRT posts after day 43, and our 1-month post-quit date outcome survey was completed around day 45 due to delays in reaching participants. Of the 52 182 total posts, to find the NRT posts, we conducted a keyword search to look for relevant terms like nicotine, nicotine replacement, NRT, patches, gum, or lozenges. We identified 1936 posts pertaining to NRT.

We used an inductive approach consistent with grounded theory to identify different themes that participants expressed about NRT in their posts to their support groups.^25^ Specifically, two researchers first familiarized themselves with the user posts by reading through the collected data and then independently identified content codes pertaining to NRT. After this the two researchers worked collaboratively to organize the codes into content categories, similar to grounded theory theme-building, and to determine whether each content category reflected a positive or negative sentiment.^26^ User posts were continually checked against the created content categories, and once all NRT-related posts could be adequately described, ie, no new information could be obtained from the data, it was determined that saturation was achieved.^25^ Overall, 12 mutually exclusive and collectively exhaustive content categories were identified that, as a set, could fully describe the NRT posts, of which 2 were deemed to be positive and 10 negative in valence.

Next, the same two researchers independently coded the content of the 1936 NRT-related posts in the dataset, using the 12 pre-identified content categories. Disagreements were resolved by a third independent researcher. After all posts had been content coded, the two original researchers worked together collaboratively to combine posts that were on very similar topics but too infrequent to allow for meaningful analyses, leaving us with 9 final content categories. Positively valenced posts about NRT working or reducing cravings were grouped together as effectiveness posts. Negatively valenced posts about mouth, stomach or skin irritation or aches or pains were grouped together as irritation posts. Two infrequent but strongly valenced negative posts, about NRT fears and NRT being too strong, were combined and labeled as miscellaneous negative. We calculated coding reliability in two ways. First, percent agreement is listed in Table 1 for each type of NRT post. This refers to the number of agreements between coders divided by the total number of times a code was assigned. Second, Cohen’s kappa was calculated to be a value of .88.

**Table 1.** Examples of NRT Posts, Coding Reliabilities and Counts.

Types of NRT Posts	Examples of NRT Posts	Percent Agreement (%)^a^	Post Counts
1. Effectiveness^b^	Aggregation of those below	83.33	671
It works^b^	“I only had one [cigarette] today. Patch works really good.”	83.15	484
Reduces cravings^b^	“Oh when strong urges hit use your gum or lozenge. It will save you!!”	83.82	187
2. Poor sleep or dreams	“I think the patches are disrupting my sleep. I am having weird dreams.”	94.91	272
3. Patch won’t stick	“Is anyone having problems with the patches not sticking? Can’t keep mine on at all help”	94.27	225
4. Irritation	Aggregation of those below	78.91	398
Mouth or stomach	“The gum makes me feel like the back of my throat is on fire!”	78.43	198
Ache or pain	“I get muscle soreness on which ever arm that I am wearing the patch.”	70.00	96
Skin	“I have scabs and a round circle on one arm from the patches!”	90.65	104
5. No longer needed	“I do not use patches anymore. I couldn’t tell the difference when I forgot them one day”	78.26	109
	“No patch again today!!! I’m holding steady. Rocking this so far!”		
6. Prefer alternative	“I use mints and straws instead to keep mouth busy…”	76.60	85
	“I’m not using the gum anymore. I switched to regular gum and it seems to do the same thing for me.”		
7. Ineffectiveness	“The patches aren’t working that great for me”	73.97	60
	“Are the patches working for you? I’ve tried them with no luck…”		
8. Dislike	“Tried the lozenges - don’t like them.”	73.91	60
	“I quit wearing the patches. They just don’t agree with me.”		
9. Miscellaneous negative	“I feel like the patch is making me floaty in the head.”	65.15	56
	“The side effects scared me and all that nicotine at once really scared me.”		

^a^Percent agreement is determined by dividing the total number of agreements by the sum of total agreements and disagreements for each type of NRT post. Cohen’s kappa was calculated to be .88.

^b^Indicates a positive post.

### Statistical Analyses

To compute descriptive statistics for our interval variables we calculated means, standard deviations, and when appropriate, medians and interquartile ranges. For our dichotomous (0,1) variables we calculated percentages. We used bivariate correlations to examine relationships among the NRT usage measures and smoking abstinence. Finally, we compared participants in our analysis sample (those who posted about NRT) to those missing from the sample (those who did not post about NRT); we used chi-square tests for binary scaled demographics (eg, gender), and t-tests for interval scaled demographics (eg, age). Relationships between (1) NRT posts and NRT usage (used at least once, use occasions, or used daily for full month), and (2) NRT posts and smoking abstinence were analyzed using generalized estimating equation (GEE) models in SPSS version 26, with the covariates of age, gender, ethnicity, education, marital status, employment status and cigarettes per day. The outcomes of NRT used at least once, NRT used daily for full month, and smoking abstinence were dichotomous (0,1), while the outcome of NRT use occasions was interval. GEE models were used to account for participants being in 36 support groups. In the analyses that used NRT posts as the predictor, all post content codes were included simultaneously in the GEE models along with the covariates. We used additional GEE models to relate the demographics listed above to NRT usage, including all demographics simultaneously.

## Results

### Demographics and NRT Usage

In our analysis sample of 438 support-group participants who posted at least once about NRT, the mean age was 39.45 (SD 9.53), 81.1% (355/438) were female, 82.5% (358/434) were non-Hispanic white, 60.1% (262/436) were married or with a significant other, 57.1% (249/436) were employed, and 67.6% (296/438) had some college. On average, participants had smoked 17.16 (SD 7.16) cigarettes per day before the current quit attempt (see Table 2).

**Table 2.** Demographics of Our Analysis Sample and Comparison to Participants Who Could Not Be Included in Our Sample.

Demographic	Those who posted on NRT; our analysis sample (n = 438, 60.83%)	Those who did not post on NRT; thus missing from the analyses (n = 282, 39.17%)	Test statistic comparing the two groups
Age	39.45	39.02	F = .34, P = .559
Gender (female)	355/438 (81.05%)	223/282 (79.08%)	χ^2^ = .42, P = .516
Ethnicity (non-Hispanic white)	358/434 (82.49%)	222/279 (79.57%)	χ^2^ = .95, P = .329
Marital status (married)	262/436 (60.09%)	156/278 (56.12%)	χ^2^ = 1.11, P = .293
Employment (employed)	249/436 (57.11%)	170/278 (61.15%)	χ^2^ = 1.14, P = .285
Education (some college)	296/438 (67.58%)	184/282 (65.25%)	χ^2^ = .42, P = .517
Cigarettes per day	17.16	17.95	F = 1.90, P = .168

*Note*. We used chi-square tests to compare the two groups (analysis sample vs missing) on binary scaled demographics, and t-tests to compare them on interval scaled demographics. Additional analyses conducted similarly showed that, among participants who posted on NRT, those who reported their NRT usage (n = 339) did not differ significantly from participants who failed to report NRT usage (n = 99) on age (*P* = .103), gender (*P* = .071), ethnicity (*P* = .579), marital status (*P* = .321), employment status (*P* = .230), education level (*P* = .081), or cigarettes per day (*P* = .085). All these analyses were bivariate. Percentages in the column heads were calculated based on n/720, referring to all treatment participants.

Among participants who posted about NRT (n = 438), 339 reported their NRT usage at 1-month post-quit date. We found that 96.17% (326/339) had used NRT at least once in the past month, 70.21% (238/339) had used NRT at least once in the past week, and 45.72% (155/339) had used NRT daily for the full month as directed. The mean number of use occasions in the prior week (7 days) was 6.91 (SD 11.31) which included patches and gum or lozenges. We also found that our NRT usage measures were themselves related but only moderately. NRT used at least once in the past week related to past-week NRT use occasions (r = .40, *P* = .001) and to NRT daily use for the full month (r = .54, *P* = .001). Also, past-week NRT use occasions related (r = .41, *P* = .001) to NRT daily use for the full month.

Several demographic variables related to NRT usage at 1-month post-quit date. Women were less likely to have used any NRT in the past week (B = −1.12, *P* = .007), and were less likely to have used NRT daily for the full month (B = −1.04, *P* = .001) compared to men. Employed participants reported more use occasions in the past week (B = 2.56, *P* = .034) than non-employed participants. More cigarettes per day at baseline was associated with any NRT use within 1 month (B = .13, *P* = .018) and more use occasions in 1 month (B = .24, *P* = .010).

### NRT Posts Related to NRT Usage

**Figure 1.**
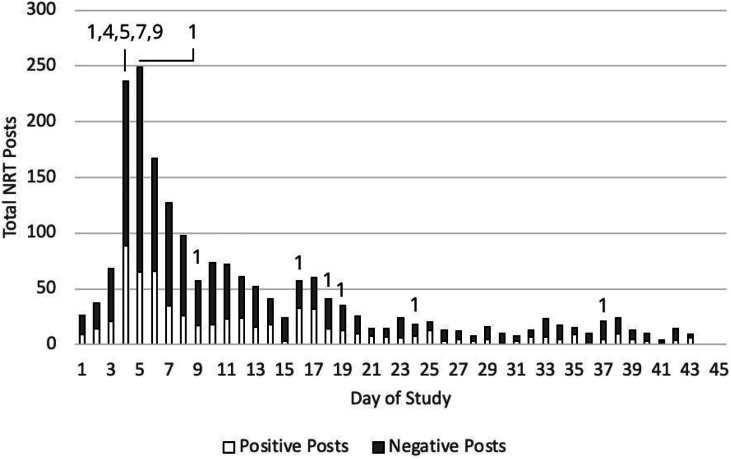
Count of NRT posts by day for the 36 online tobacco-cessation support groups (n = 438). Note: Positive NRT posts discussed effectiveness. Negative NRT posts encompassed the remaining categories. Numbers on top of the bars indicate the type of NRT info we provided to all participants via email or post: 1 about effectiveness, 4 irritation, 5 no longer needed, 7 perceived ineffectiveness, and 9 miscellaneous negative (see Appendix B for details).

During the first 45 days of participation in their tobacco-cessation support groups, the 438 participants in our analysis sample produced 52, 182 total posts to their groups, averaging 119.14 posts per person (SD 213.98, Median 66, interquartile range [IQR] 93). Of the 52 182 total group posts, 1936 (3.7%) were about NRT, averaging 4.42 NRT posts per person (SD 4.69, Median 3, IQR 4). Negative NRT posts made up 65.34% of NRT posts (1265/1936), with positive NRT posts making up 34.66% (671/1936). On average, each online support group produced 35.14 negative and 18.64 positive posts about NRT. Negative NRT posts exceeded positive ones on 79.07% (34/43) of the days with NRT posts. The NRT posts primarily occurred early in the study, when participants started using their free NRT, or even beforehand, with 47.00% (910/1936) of the NRT posts occurring within the first week and 70.45% (1364/1936) occurring within the first two weeks. Figure 1 shows the count of NRT posts per day by valence as well as the type of NRT information provided by day.

At 1-month post-quit date, NRT effectiveness posts related positively to NRT used at least once in the past week (B = .15, *P* = .023) and NRT daily use for the full month (B = .25, *P* = .001). NRT no longer needed posts related negatively to NRT used at least once in the past week (B = −.37, *P* = .045), NRT use occasions in the past week (B = −1.10, *P* = .016), and NRT daily use for the full month (B = −.53, *P* = .009). NRT dislike posts related negatively to NRT used at least once in the past week (B = −.66, *P* = .005), NRT use occasions in the past week (B = −1.86, *P* = .018), and NRT daily use for the full month (B = −.56, *P* = .044). Nicotine replacement therapy effectiveness posts related positively to smoking abstinence (B = .27, *P* = .002), as did NRT no longer needed posts (B = .61, *P* = .005). Posts about poor sleep or dreams, patch won’t sick, irritation, ineffectiveness or miscellaneous negative did not relate to our NRT usage measures or smoking abstinence. These results are summarized in Table 3.

**Table 3.** GEE Results Relating NRT Posts to NRT Use and Smoking Abstinence.

	NRT Used at Least once in Past Week (n = 339, 77.40%)		NRT Use Occasions in Past Week (n = 339, 77.40%)		NRT Daily Use for Full Month (n = 339, 77.40%)		Smoking Abstinence (n = 403, 93.15%)	
Types of NRT Post	B (1,322)	*P*-value	B (1,322)	*P*-value	B (1,322)	*P*-value	B (1,388)	*P*-value
1. Effectiveness^a^	.15	.023	.89	.055	.25	.001	.27	.002
2. Poor sleep or dreams	.01	.982	−.45	.233	−.02	.859	.13	.243
3. Patch won’t stick	.21	.084	−.57	.139	.06	.582	−.09	.413
4. Irritation	−.05	.631	.32	.420	.06	.545	−.03	.785
5. No longer needed	−.37	.045	−1.10	.016	−.53	.009	.61	.005
6. Prefer alternative	.16	.462	.65	.443	−.26	.209	−.19	.356
7. Ineffectiveness	−.06	.844	−1.74	.118	.02	.947	−.14	.626
8. Dislike	−.66	.005	−1.86	.018	−.56	.044	−.43	.091
9. Miscellaneous negative	−.09	.807	−1.58	.186	.55	.155	.24	.348

*Note*. Outcomes were assessed at 1-month post-quit date.

^a^Indicates positive post content. Percentages in the column heads were calculated based on n/438, the number of participants who posted about NRT (our analysis sample).

### NRT Usage and Smoking Abstinence

In our analysis sample of 438 support-group participants who posted at least once about NRT, 403 reported their smoking abstinence at 1-month post-quit date. (We obtained relatively high response rates on abstinence because this was our main priority and the first question asked.) Our analysis showed that 155/403 (38.46%) of them were abstinent from cigarettes. Moreover, NRT use occasions in the past week (r = .17, *P* = .002) and daily NRT for the full month (r = .18, *P* = .001) related to smoking abstinence; but use of NRT at least once in the past week did not relate to smoking abstinence (r = .07, *P* = .226).

## Discussion

### Summary

The current study contributes to the literature by examining, in a social media-based tobacco-cessation intervention that provided free NRT, the content of participants’ NRT posts and how their posts related to their NRT usage and smoking abstinence. In our tobacco-cessation intervention which provided treatment participants with free combination NRT and a private social media-based support group, nearly all (96.17%) participants reported using NRT at least once at 1-month post-quit date. Also, most (70.21%) used NRT during the past week, but less than half (45.72%) used NRT daily for the full month as recommended. Nicotine replacement therapy usage in our study was comparable to what we saw in the literature. The mean number of NRT use occasions in the past week was 6.91 including patches and gum or lozenges. Both past-week use occasions and past-month daily use of NRT related to smoking abstinence.

To better understand NRT usage, we examined the valence and content of participants’ NRT-related posts to their online support groups. Posts about NRT made up a minority (3.7%) of total posts, but most of the NRT-related posts were negative (65.34%), and posts about disliking or no longer needing NRT related to poor NRT adherence, while posts about NRT’s effectiveness related to better NRT adherence and smoking abstinence.

We also found that 70% of participants’ NRT-related posts occurred within the first 2 weeks of group start. This finding indicates it is important to encourage and instruct on NRT usage at the earliest stages of tobacco-cessation support groups. Participants began posting about NRT before they were even told to start it on day 4, and therefore efficacy information, usage instructions, and side effect mitigation information should be distributed as soon as participants start setting quit dates. We emailed participants a detailed NRT usage guide, responded to individuals’ NRT concerns and provided periodic posts and smokefree.gov links promoting NRT use; but, based on our findings, some of this information should have been sent earlier and we should have placed more emphasis on addressing NRT concerns not just NRT efficacy. While posts about specific NRT side effects did not relate to usage, general dislike of NRT lowered usage and, to counter that general dislike, it should be useful to proactively provide solutions to common NRT problems. It is also important to stress NRT efficacy early and repeatedly, as we did, because efficacy perceptions related to both NRT usage and smoking abstinence. NRT no longer needed posts related negatively to NRT usage but positively to abstinence indicating that participants who stopped NRT because they felt ready to do so tended to successfully abstain.

An additional result was that past-week NRT use occasions and daily use for one month related positively to smoking abstinence, while NRT used at least once in the past week did not relate to abstinence. This result indicates that measuring adherence to the NRT treatment protocol by asking about one time use in the past week is inadequate. Continued, repeated usage of NRT should be measured and encouraged throughout the tobacco-cessation process.

The social media-based support groups in the current study were not moderated; hence, participants’ posts about not liking NRT did not generate a professional response or advice to continue use. As a result, the data provide a novel birds-eye-view into the naturalistic experience of using NRT and participants’ perspectives of NRT in relation to their adherence. Future interventions might want to use personalized counseling or automated feedback, eg, from a Chatbot, to encourage better NRT adherence.

Study limitations include reliance on self-reported smoking abstinence and NRT adherence and missing data on some participants. Study strengths include the unique nature of capturing participant exchanges via a social media-based support group and the ability to link participant posts to NRT usage and smoking abstinence.

### Implications

This research contributes to the currently limited body of work concerning how users’ perceptions of NRT may affect their NRT usage and smoking abstinence. In prior studies providing free NRT, many participants did not use the NRT daily as recommended, especially after two weeks. Adherence to NRT usage guidelines should be closely monitored and actively promoted. In addition, NRT usage measures should consider length and frequency of use at various time points and look beyond the first few or even several weeks.

In the current study of social media-based support groups for tobacco cessation, posts about dislike of NRT related to lower NRT usage, whereas posts about NRT effectiveness related to higher NRT usage and greater smoking abstinence among those who made those posts. Hence, tobacco-cessation studies or support groups which offer free or low cost NRT or otherwise encourage its use should highlight the positives (eg, it works and reduces cravings) and counter the negatives (eg, dislike, irritation, perceived ineffectiveness) to better help people quit. To highlight the positives of NRT, researchers can stress the 50% - 70% greater likelihood of quitting tobacco while using NRT, while also telling participants that combining a form of counseling or support program for quitting while on NRT can more than double the chances of success.^1-3^ Numerous NRT guides and videos are available on Smokefree.gov, many of which were added to the website after our intervention. Combatting negative perceptions of NRT can be done by promoting proper usage of the product to minimize side effects. Negative side effects of NRT, while generally minimal, can occur.^27^ Therefore, researchers should proactively inform participants of common side effects and how to resolve them. Common gastrointestinal side effects of NRT can usually be resolved by the park and chew method for nicotine gum or lozenge, which reduces the amount of nicotine that is swallowed. Insomnia, while a common side effect of nicotine withdrawal generally, can be combatted by removing nicotine patches before sleep. Skin irritation can be reduced by moving the nicotine patch placement site around the body, or by stepping down NRT dosage. By actively stressing the effectiveness of NRT while simultaneously helping to reduce common side effects from NRT, researchers should be able to reduce the general feeling of dislike of NRT among participants that related to lower usage. These strategies can be utilized in tobacco-cessation interventions that provide or promote NRT, and also to respond to NRT-related social media posts in social media-based intervention programs or even in general settings.

## Conclusions

Non-adherence has been an issue in past tobacco-cessation studies that provided free NRT. In the current study, the majority of participants’ NRT posts were negative in valence, which related to posters being NRT non-adherent, that is, not using NRT daily for the entire month as recommended. Yet, a substantial number of participants’ NRT posts were positive in valence, and in particular they remarked on NRT efficacy, which related to the posters using NRT more, being NRT adherent, and abstaining from smoking. Overall, our results indicate that social media posts about NRT should, whenever possible, be monitored and responded to with clinically relevant advice promoting NRT usage. Moreover, researchers who provide free NRT in tobacco-cessation programs should proactively try to counter participants’ negative perceptions about NRT and stress NRT efficacy to encourage medically compliant usage and improve abstinence rates.

**So What?**

**What Is Already Known on This Topic?**

Usage of free study-provided NRT in quit-smoking programs is routinely measured, but often based on participants using the NRT at least once; and adherence is much lower using more comprehensive and rigorous measures.

**What Does This Article Add?**

In the current study, nearly all (96.17%) participants reported using NRT at least once at 1-month post-quit date, most (70.21%) used NRT during the past week, but less than half (45.72%) used NRT daily for the full month as recommended. Most participants’ NRT posts were negative in valence and related to non-adherence. Yet, a substantial number of NRT posts were positive in valence, and in particular remarked on NRT efficacy, which related to greater use of NRT, NRT adherence, and abstaining from smoking.

**What Are the Implications for Health Promotion Practice or Research?**

Strategies are needed to address users’ dislike of NRT and strengthen perceptions of NRT efficacy, especially on social media where posts may be amplified.

## Supplemental Material

Supplemental Material - Understanding Hesitation to Use Nicotine Replacement Therapy: A Content Analysis of Posts in Online Tobacco-Cessation Support Groups Click here for additional data file. Supplemental Material for Understanding Hesitation to Use Nicotine Replacement Therapy: A Content Analysis of Posts in Online Tobacco-Cessation Support Groups by Connor Phillips, Cornelia Pechmann, Douglas Calder, and Judith J Prochaska in American Journal of Health Promotion
