# Supplementary material for: Understanding Hesitation to Use Nicotine Replacement Therapy: A Content Analysis of Posts in Online Tobacco-Cessation Support Groups
Source: Am J Health Promot. 2022 Jul 11;37(1):30–8. doi: 10.1177/08901171221113835 (PMC9755690; doi:10.1177/08901171221113835)
Supplement: Supplemental Material - Understanding Hesitation to Use Nicotine Replacement Therapy: A Content Analysis of Posts in Online Tobacco-Cessation Support Groups [file sj-pdf-1-ahp-10.1177_08901171221113835.pdf]

Online Appendix A. Nicotine replacement therapy (NRT) usage in past tobacco-cessation studies

Study	Sample Demographics	NRT Type	NRT Duration	NRT Usage Measurement	NRT Usage Outcome
A. Dual NRT					
1. Hajek et al., 2019	U.K. adult smokers in tobacco-cessation study	Patch, gum, lozenge, inhaler	4 weeks	Median days used in past month, at 1 month	24 days out of 30
				Median days used in past week, at 1 month	6.5 days out of 7
2. Kerr et al., 2019	Minnesota enrollees in tobacco-cessation program	Patch, gum, or lozenge	2 weeks	How much used, at 1 month	44% all, 48% some, 8% none.
3. Voci et al., 2016	Canadian tobacco-cessation program attendees	Patch, gum, or inhaler	10 weeks	How much used, at 2.5 months	20% all, 28% most, 47% some, 5% none.
4. Tworek et al., 2009	Maine quitline callers	Patch and gum	8 weeks+	Number of days used, at 3 months	10% >60 days out of 60+, 42% 31-60 days, 26% 15-30 days, 22% 1-14 days.
B. Patch Only					
1. Kushnir et al., 2017	Canadian adult smokers in tobacco-cessation study	Patch	5 weeks	How much used, at 2 months	11% all, 48% some, 41% none.
2. Cummings et al., 2010	New York quitline callers	Patch	4 weeks	Used at least once, and number of days used, at 3-7 months	1% >43 days out of 48, 3% 29-42 days, 13% 15-28 days, 43% 8-14 days, 25% 1-7 days, 15% 0 days.
" "	" "	Patch	8 weeks	Used at least once, and number of days used, at 3-7 months	37% >43 days out of 48, 17% 29-42 days, 16% 15-28 days, 9% 8-14 days, 18% 1-7 days, 3% 0 days.
3. Bush et al., 2008	Oregon quitline callers	Patch	2 weeks free	% who bought more, and mean days used, at 6 months	54% bought more patches; 28 days used.
4. Cummings et al., 2006	New York quitline callers	Patch	1 week	Used at least once, and mean days used, at 4 months	78% used at least once; 5 days out of 7.
" "	" "	Patch	2 weeks	Used at least once, and mean days used, at 4 months	81% used at least once; 9 days out of 14.
" "	" "	Patch	6 weeks	Used at least once, and mean days used, at 4 months	89% used at least once; 21 days out of 42.

Note – Studies listed by NRT type, then in chronological order starting with the most current. Cummings et al. 2006 and

2010 reported other similar NRT usages for other treatment scenarios.

Online Appendix B. NRT-related messages provided to participants by study day

Day	Type of NRT Message	NRT info category	Smokefree.gov Link or Discussion Post Content
4	NRT email	1, 4, 5, 7, 9	Nicotine replacement therapy guide, including effectiveness statistics, dosage information, dropping down the dosage if too strong, and ways to avoid skin and stomach irritation
5	Smokefree.gov link sent via email	1	Guide with information on NRT effectiveness and ways to create a quit plan: https://smokefree.gov/quitting-smoking/prepare-quit
9	Smokefree.gov link sent via email	1	Guide with information on smoking triggers and how NRT can help: https://smokefree.gov/challenges-when-quitting/cravings-triggers/know-your-smoking-triggers
16	Discussion post for online support group	1	Are you using nicotine gum/lozenges? Nicotine patch? How often? Does this help you manage cravings? Share your experience with your group!
18	Discussion post for online support group	1	How do you keep nicotine patches and gum/lozenges handy? Do you keep them in a certain place, and if so, where?
19	Smokefree.gov link sent via email	1	Guide with information on managing cravings and how NRT can be used effectively: https://smokefree.gov/challenges-when-quitting/cravings-triggers/how-manage-cravings
24	Discussion post for online support group	1	How do you remind yourself to put on a new nicotine patch each day? Share your ideas with the group!
37	Discussion post for online support group	1	What step are you on with your nicotine patches and how are they working? Share your answer with your group!

Note - Numbers indicate the type of NRT info we provided to all participants via email or post: 1 about effectiveness, 4 irritation, 5 no longer needed, 7 perceived ineffectiveness, and 9 miscellaneous negative.

Online Appendix C. NRT-related emails for participant concerns

Type of Message	Message Text
Nicotine Replacement Therapy Overview	The Tweet2Quit program provides you with nicotine patches and gum/lozenges because they will increase your chances of quitting smoking. It is very important that you use the nicotine patches and gum or lozenges daily for 8 weeks, starting on your quit date (or the night before). Studies show you are 50-70% more likely to quit if you use these products. Use at least one product, preferably two, to give yourself the best possible chance of quitting. Start using your nicotine patches and gum on your quit date and continue use until you run out. Even if you don't feel withdrawal symptoms, please continue using the patch for the full amount of time. The nicotine patches and gum/lozenges will help to wean you off cigarettes, but follow the instructions provided to avoid overdose. If the nicotine patch seems too strong, drop down to the lower dose. While using the patch, limit your use of nicotine gum/lozenges to 12 pieces per day. Nicotine gum isn't like regular gum, so please check out our linked guide for a helpful video on how to use it. If you chose the lozenges instead, please read this linked guide for proper use. If you chew the gum or suck the lozenge too much, you could get a stomachache and it will taste terrible. Instead chew or suck the product briefly until there is a slight tingling, then park it in your cheek. Skin reactions are one of the more common side-effects of nicotine patches. You may notice a little redness, itching or burning under the patch which usually settles soon after the patch is removed. In more severe cases there may be an irritating rash or swelling. Make sure you apply the patch to a different site every day, without reusing the same site for at least a week. You can also spray a steroid (corticosteroid) onto the skin or directly onto the patch before applying it. Steroids reduce inflammation and this may prevent a reaction from occurring. The spray should not affect the adhesiveness of the patch.
Nicotine Patch Quality	We are sorry that you are having difficulties with the Habitrol nicotine patches. Please visit the manufacturer's website for more information. We have not heard complaints about this brand from other program participants. This is a popular brand that is widely available and is regulated by the FDA to have the same potency as other brands on the market. If you have any specific questions, you can try contacting the company. Unfortunately, this is the only brand that we can provide.
More Nicotine Patches	Thank you for your inquiry. Please feel free to buy more of any step of patches and use them for as long as you wish, before dropping to the subsequent lower dose. However, we cannot provide additional patches; our budget and research protocol won’t allow us to do this. We can only provide the recommended, standard number of patches.
Nicotine Patch Not Sticking	If you are having trouble with the nicotine patch sticking, please try taping the patches on with first aid or skin-safe tape. Be sure the area is clean, dry, and lotion-free! Please let us know if you have any further questions.

Nicotine Patch High Dosage	In some cases, it is possible to have a reaction to the highest dosage of nicotine patches. Please try the next step down, which is Step Two - 14mg patches. If this still causes problems, further reduce to the Step Three - 7mg patches, or contact us again.
Nicotine Patch Health Issue	We advise to stop using both the nicotine patches and gum as you are experiencing these symptoms while using them. At it seems these products are causing these problems, we advise that you consult a doctor about using non-NRT (Nicotine Replacement Therapy - gum, and patches) methods of quitting smoking. Additionally consult a doctor if these sores/bleeding are still present so that they can be treated. If you have any other questions about this, or need any further help, please let us know. We hope you still use your group as a helpful tool to quit smoking as this can still be very beneficial even without the patches or gum.
Nicotine Patch/Gum Addiction	There is an extremely low risk of becoming addicted to the nicotine patch. Rarely, people may have some difficulty reducing gum use. Nicotine is an addictive drug. It is the drug's speed of delivery, however, that influences its addiction potential. Nicotine reaches the brain from smoking a cigarette in 8 to 10 seconds - no delivery source matches that - not even intravenous use. The rapid spike up and then down of nicotine delivery from a cigarette leaves the smoker wanting more. The patch in comparison provides a slow and more steady level of nicotine, which is not dependence producing. The gum is far closer to the patch than the cigarette and of course doesn't deliver the CO and other toxins that you get from a combustible cigarette.
